# Supplementary material for: Epidemiological Analysis of Fungal Infection Disease in Pediatric Population: Focus on Hospitalization from 2007 to 2022 in Veneto Region in Italy
Source: Pathogens. 2025 Jan 18;14(1):93. doi: 10.3390/pathogens14010093 (PMC11768092; doi:10.3390/pathogens14010093)
Supplement: Supplementary file 1 [file pathogens-14-00093-s001.zip › pathogens-3414041-Supplementary File S1.pdf]

SUPPLEMENTARY

CODES ICD-9-CM DIAGNOSIS

- 1118 - 1119, 1179 Dermatomycosis
- 1173 Aspergillosis
- 4846 Pneumonia due to *Aspergillus*
- 1177 Zygomicosis infection
- 1179, 71160-71169 Other Mycosis
- 118 Mycosis from facultative mycosis
- 4847 - 4848 Pneumonia due to systemic mycosis
- 11281-11289, 7717 Neonatal *Candida* infection
- 6910 Diaper *Candida* infection
- 1120-1129 Candidiasis
- 38015 Mycotic otitis

CODES ICD-9-CM COMORBIDITIES

- Prematurity: 76510-76519; 76400 -76419
- Solid organ transplantation: 99680-99687; V420, V421, V426, V427, V429, V4289
- Immunocompromised, onco-hematological: V5811, 20020-20028; V1081; 20400, 20401; 20490; 20491; 20500; 20501; 20590
- Cystic fibrosis: 27702, 27709
